# Independent mobility in relation to weekday and weekend physical activity in children aged 10–11 years: The PEACH Project

**DOI:** 10.1186/1479-5868-6-2

**Published:** 2009-01-07

**Authors:** Angie S Page, Ashley R Cooper, Pippa Griew, Laura Davis, Melvyn Hillsdon

**Affiliations:** 1 Department of Exercise, Nutrition & Health Sciences, University of Bristol, Bristol, UK

## Abstract

**Background:**

Children's independent mobility has fallen in recent years and may in part explain reported declines in physical activity in young people. This cross-sectional study investigated whether independent mobility in boys and girls was related to objectively measured physical activity.

**Methods:**

Thirteen hundred and seven 10–11 year old boys and girls from 23 schools in a large UK city took part. Measures included objectively recorded physical activity (accelerometer (Actigraph GT1M)), height (m) and weight (kg), a newly developed scale for local (Local-IM) and area independent mobility (Area-IM), minutes of daylight after school, level of neighbourhood deprivation and pubertal status.

**Results:**

Boys had greater Local-IM, Area-IM and physical activity (average weekday and weekend counts per minute) compared to girls. In linear regression analyses (adjusting for minutes of daylight after school, neighbourhood deprivation, pubertal status and body mass index) higher scores for Local-IM and Area-IM were significantly (p < 0.01) related to higher levels of physical activity on weekdays for boys and girls. For weekend physical activity, only Local-IM in girls remained significant (p < 0.05) in the model.

**Conclusion:**

Independent mobility appears to be an important independent correlate of weekday physical activity for both boys and girls.

## Background

There is growing evidence that children's independent mobility has been declining in recent years. For example, the proportion of 10 to 11 year old children travelling unaccompanied to school in the UK fell from 94% in 1970 to 54% in 1990 [1] and 47% in 1998 [2]. Data from the UK National Travel Survey [3,4] show that in 2002 children were taking 4.7% fewer trips outside the home than in 1985/86 and that the proportion of trips undertaken on foot had declined from 47% to 32%, with an increase in the use of cars from 35% to 56%. These findings are supported by qualitative data which found that the numbers travelling to school alone at the age of 10–11 had fallen from approximately 40% for those born in 1932–1941 to 9% for those born in 1990–1991 [5]. Thus, it appears that children may be spending less time unsupervised outside the home and when they do go out, are more likely to travel by car. Similar trends have been reported in other countries. For example, in the U.S. children's active commuting to school declined by 37% between 1977 and 1995 [6], and in Italy 71% of 7–12-year-old children are always accompanied by adults on journeys to and from school [7]. In Australia, 32% of 8–12 year old children report travelling < 100 m from home without an adult [8].

Reductions in independent mobility and increased car travel may be due to a perceived threat from traffic, molestation, distance to destination, convenience and opportunity for social interaction between parent and child [1,9-11]. Other significant social trends, which may indirectly influence children's independent mobility, include changing family structure, greater use of structured childcare and change in parental working patterns [12]. Children with greater independent mobility (freedom to move around unsupervised by adults) are reported to interact more with their peers, both indoors and outdoors, whilst children with lower independent mobility interact less with adults beyond the confines of the family [13]. Greater independent mobility has also been related to higher acquisition, processing and structuring of environmental knowledge in 8–11 year olds [14]. Conversely, low levels of independent mobility can negatively influence children's emotional, social, and cognitive development [15]. Gingsburg [16] proposes that reduced interaction with the environment limits opportunity for play, particularly child-centred play (unsupervised by adults) which is important for the development of a range of skills including negotiation and group working. These findings suggest that children with lower independent mobility are less connected to their environment which could lead to decreased interaction with and increased fear of their local neighbourhood [13].

Low independent mobility may lead to decreased levels of physical activity and increased sedentary activities, putting children at risk of developing obesity [17,18]. Adolescent females who spend more time unsupervised after school have been reported to be more physically active than those who do not spend as much time unsupervised after school [19]. It seems important therefore to further investigate whether higher levels of independent mobility are related to increased physical activity in children.

Children's independent mobility has been operationalised in different ways. It has been described as 'territorial range' based on the distance from children's homes to places they visit when playing [20] and as ' play participation' (whether children play outside and how often) [14]. Hillman and others [1,8] have described independent mobility in relation to 'license' or degree of independence to move around in the neighbourhood. Some studies have attempted to quantify the level of children's mobility within a certain period, using for example mobility diaries [15]. These approaches capture important distinct elements of independent mobility but conceptual clarity and appropriate child-based measures are lacking [21]. Despite the increased interest in perceptions of the environment and physical activity in recent years and the now routine assessment of neighbourhood characteristics such as access to recreational facilities, transport infrastructure, urban form, and neighbourhood conditions [22,23], the concept of independent mobility in relation to physical activity has received limited attention. Where independent mobility has been included as a measure it has largely been considered in small samples [8] or related to self-reported rather than objective measures of physical activity [2,7,13].

This study sought to investigate whether self-reported independent mobility (IM) was related to objectively measured weekday and weekend physical activity in children aged 10–11 years. This study also attempted to account for a wide range of covariates that may confound the relationship between independent mobility and physical activity, an acknowledged weakness in this area of research [22,24]. Independent mobility is defined here as 'the opportunity for children to move around in their neighbourhood unaccompanied by an adult.'

## Methods

This study is based on the baseline data from the PEACH project (Personal and Environmental Associations with Children's Health). The PEACH project is a longitudinal study designed to investigate the environmental and personal determinants of physical activity, eating behaviours and obesity in young people as they transition from the final year of primary school (aged 10 to 11 years) to the first year of secondary school (11 to 12 years).

### Participants

Thirteen hundred and seven year 6 children were recruited from 23 of the 72 state funded primary schools within a large UK city between September 2006 and July 2008. These primary schools were selected as they had the highest transition rates (> 40%) to one of eight urban state funded secondary schools selected on the basis of the Index of Multiple Deprivation (IMD) and geographic location to represent Bristol. The Index of Multiple Deprivation is a composite score based on seven categories of deprivation (income, employment, health and disability, education skills and training, housing, and geographical access to services) [25]. IMD scores are based on the post-code of the school and a lower score indicates a higher level of deprivation. Compared to IMD values for England, the primary schools in this study were located in relatively deprived areas (4 schools were ranked in the lowest decile for IMD, 4 in 2^nd ^decile, 5 in the 3^rd ^decile, 1 in 4^th ^decile and 9 in deciles 6–10). Schools were also located in relatively deprived neighbourhoods compared to the local region (Avon) with 13 of the 23 schools ranked in the lowest two deciles for IMD and only 7 ranked in deciles 6–10. Only one primary school approached declined to take part in the study.

### Procedure

Schools were contacted by phone and/or by letter to invite them to take part in the study. For those schools who agreed to participate, Year 6 school children and teachers were briefed about the study and provided with an information pack to take home. On measurement days, only children who provided written parental consent were invited in small groups (4–6 children) to take part. Children had their height and weight measured in a private room by the researcher and then completed a computerised self-report questionnaire with a researcher nearby if help was required. Children were given an accelerometer to take home and instructed to wear it for seven days and then bring it back to school. Children were provided with a certificate and a small toy (e.g. Frisbee or ball) for taking part and schools were provided with a poster describing a class-based summary of, for example, physical activity levels of boys versus girls, current physical activity guidelines and the health benefits of optimal physical activity levels. This study was carried out in accordance with the Declaration of Helsinki and ethical approval was provided by University of Bristol Ethics Committee (Ref: 009/006).

### Measures

#### Dependent variables

##### Physical activity

Physical activity was measured using an accelerometer (ActiGraph GT1M; ActiGraph, FL, USA), a widely used objective measure of physical activity in children [26]. Children were instructed to wear the accelerometer on a belt around their waist during waking hours for seven consecutive days and the instruments were programmed to record data every 10 seconds. After downloading data from the actigraph, data reduction was initially carried out using the MahUffe software . Periods of 60 continuous epochs (10 minutes) with 0 values were excluded and only weekdays with at least 480 minutes of registered time were considered in analyses. The dependent variables used were average counts per minute for weekdays and weekends.

#### Independent variables

##### Independent mobility (IM)

Independent mobility was assessed using eleven questions, which were part of a self-completed computerised questionnaire. These eleven questions were hypothesised to represent children's independent mobility to visit a range of destinations in the local and wider neighbourhood. Children were asked '*How often are you allowed to go to the following places on your own or with friends (without an adult)*?' For each destination (local shops, big shopping centre, park or playground, sports centre, swimming pool, library, school, cinema, friend's house, amusement arcade, bus stop or train station) children responded using the scale never (1), sometimes (2), often (3), and always (4). An additional response was also available to select 'I don't go there (5)'. These questions were based on common destinations reported in previous work [8] and on pilot data with 175 children (84 boys, 91 girls) from a large UK city. In the pilot study, the question format was piloted and additional open questions were included to allow children to report any destinations which were not in the questions provided. The eleven destinations here represented those destinations most frequently visited by children in the pilot sample.

As the items hypothesised to represent independent mobility were significantly correlated, principal components analysis (PCA) with Varimax rotation was conducted in SPSS (Version 14.0 SPSS Inc., Chicago 2004) to reduce the items into associated components. The resulting scree plot and Eigen values were inspected and interpreted and factors selected. This process resulted in two factors; 1) Area independent mobility (Area-IM: Cronbach alpha = 0.788) [26] which accounted for 36.16% of the variance and included the places likely to be some distance from the home. 2) Local independent mobility (Local-IM: Cronbach alpha = 0.703) which accounted for 10.72% of the variance in all the items and included destinations likely to be local to children's homes. PCA were similar for males and females so generic means were generated for each factor and used in subsequent analyses. The results of the principal component factor analysis are shown in Table 1. Subscale scores were weighted to account for those who did not visit each destination and recoded such that a greater score represented greater independent mobility (mean scores ranged from 1 to 4). Intra-class correlation (ICC) values [27] for these scales with a sub-sample of children (n = 46) from the same city over a two week period were ICC = 0.81 (Local-IM) and ICC = 0.78 (Area-IM).

**Table 1:** Factors obtained from principal components analyses with varimax rotation

**Item**	**Area** Independent Mobility	**Local** Independent Mobility
Sports centre	**.723**	.145
Swimming pool	**.711**	.168
Big shopping centre	**.625**	.169
Amusement arcade	**.610**	.150
Library	**.591**	.196
Bus stop/train station	**.586**	.165
Cinema	**.534**	.365
Friends House	.116	**.761**
Local shops	.235	**.708**
Park	.245	**.688**
School	.169	**.615**

**Eigenvalue**	**3.978**	**1.18**
**Variance Explained**	**36.16%**	**10.72%**

##### Daylight

Minutes of daylight available on the first day of measurement for the ActiGraph were determined from standard tables [28]. The variable used was minutes of daylight from 3 pm until sunset as an indicator of available daylight after school.

##### Level of Deprivation

The UK Index of Multiple Deprivation (IMD) 2007 score based on full home post-code was used as an index of neighbourhood deprivation for each child [25]. A lower score indicates a higher level of deprivation.

##### Pubertal status

Pubertal status was measured using the scale developed by Petersen [29] and five derived stages (equivalent to Tanner stages) were used in analyses.

##### Body mass index (BMI)

Height (m) and weight (kg) were measured using a beam scale and stadiometer (SECA), with children wearing indoor clothing, and shoes removed. BMI was calculated (weight in kg divided by height in metres squared).

Gender, date of birth and full post-code for participating children were confirmed by the Local Education Authority.

### Data analyses

Means and standard deviations were calculated for all variables except for pubertal status where frequencies were calculated. Independent t-tests were used to assess differences in mean scores between gender, and χ^2 ^were used to examine differences in frequencies for pubertal status.

After conducting univariate correlations to confirm relationships with physical activity, the independent variables Local-IM and Area-IM were entered into linear regression analysis using Stata (version 10, College Station, TX) with mean weekday and weekend counts per minute as the dependent variable. An interaction term (Local-IM × Area-IM) was also entered into the regression analysis as Local-IM and Area-IM may relate to each other as well as to physical activity. Potential confounders in the analyses were minutes of daylight after school, pubertal status, level of neighbourhood deprivation and BMI. As participants from similar schools may share some characteristics the models were adjusted for school. The significance level was set at 0.05. Due to the well documented gender differences in physical activity and independent mobility all regression analyses were carried out separately by gender [13,30].

## Results

Of the 1899 Year 6 children invited to take part in the study, 1340 provided parental consent (70.5%). Of these 33 were absent on the days of measurement. Seven participants did not complete the computerised questionnaire leaving 1300 for analysis. ActiGraph data was unavailable for 23 participants due to non-returned or broken instruments. A further 48 children had insufficient registered time for weekdays to be included in the analyses. The figure for weekends was higher with 366 children not meeting the criteria for registered time. There was no significant (p < 0.05) difference between those included and excluded in the weekday physical activity analyses for age, IMD, BMI, pubertal status or Local-IM. Participants with excluded weekday physical activity data did have significantly higher mean scores for Area-IM (2.19 (SD = 0.927) vs 1.90 (SD = 0.736), t = -2.61, p = 0.011, p = 0.37) and minutes of daylight from 3 pm to sunset (194.79 (SD = 85.89) vs 222.53 (SD = 78.99, t = -2.65, p = 0.008). Participants with excluded weekend physical activity had significantly higher mean scores for Local-IM and Area-IM (Local-IM: 3.14 (SD = 0.768) vs 3.04 (0.753), t = -2.93, p = 0.003; Area-IM: 2.04 (SD = 0.796) vs 1.87 (SD = 0.726), t = -3.54, p = 0.001) and lived in less deprived neighbourhoods (IMD score: 30.40 (SD = 18.40) vs 25.97 (SD = 17.44), t = -4.03, p = 0.001).

Descriptive statistics are presented for all participants and by gender in Table 2. Males had significantly greater levels of Local-IM and Area-IM and weekday and weekend physical activity compared to females, who had greater levels for BMI and were more advanced in terms of pubertal status. There was no significant gender difference in age, minutes of daylight available after school or index of multiple deprivation. For both males and females scores for Local-IM were greater than Area-IM.

**Table 2:** Means and frequencies of variables for all participants and by gender

	**All** **(n = 1300)**	**Males** **(n = 639)**	**Females** **(n = 661)**	**t, p**
	**Mean (SD)**	**Mean (SD)**	**Mean (SD)**	
Age (years)	10.95 (0.414)	10.96 (0.442)	10.93 (0.407)	1.18, p = 0.238
Local Independent mobility	3.04 (0.760)	3.11 (0.747)	2.97 (0.767)	3.33, p = 0.001
Area independent mobility	1.91 (0.751)	1.98 (0.708)	1.85 (0.708)	3.22, p = 0.001
Physical activity (average weekday counts per minute)	642 (191)	704 (193)	583 (170)	11.62, p < 0.001
Physical activity (average weekend counts per minute)	653 (284)	694 (279)	618 (282)	4.11, p < 0.001
Minutes daylight after school (3 pm to sunset)	196 (85.7)	194 (85.2)	197 (86.2)	-0.714, p = 0.476
Level of deprivation (IMD score)	27.29 (17.84)	27.75 (18.43)	26.85 (17.25)	0.916, p = 0.360
BMI (kg/m^2^)	18.56 (3.41)	18.18 (2.98)	18.92 (3.74)	-3.96, p = 0.001

Pubertal Status	N (%)	N (%)	N (%)	
stage 1	234 (18.0)	133 (20.8)	101 (15.3)	**χ**^2 ^= 6.82, p < 0.001
2	395 (30.4)	249 (39.0)	146 (22.1)	
3	582 (44.5.0)	229 (35.8)	353 (53.4)	
4	87 (6.7)	27 (4.2)	60 (9.1)	
5	2 (0.2)	1 (0.2)	1 (0.2)	

* P-value is for independent t-test for continuous measures and Chi-square test for pubertal status and mode of travel

BMI: body mass index, IMD:index of multiple deprivation

Pearson correlation co-efficient for all variables are presented in Table 3. Local-IM was moderately positively correlated with Area-IM. Correlations between Local-IM and weekday and weekend physical activity were generally positive but weak. Similarly Area-IM was positively, weakly, related to physical activity on weekdays and at the weekend. Pubertal status, BMI, and daylight were all weakly, positively, correlated with both Local-IM and Area-IM. IMD score was significantly correlated with Area-IM but not Local-IM. Daylight was positively significantly related to physical activity as was IMD. Both pubertal status and BMI were negatively related to weekday physical activity. As these variables were generally related to both independent mobility and physical activity, and there is no strong evidence that they are on a causal pathway between the two, they were considered as confounders in the regression analyses [31].

**Table 3:** Pairwise Pearson correlation coefficients between independent mobility, physical activity and potential confounders

	**Local-IM**	**Area-IM**	**Pubertal Status**	**BMI**	**IMD**	**Daylight**	**Weekday physical activity**	**Weekend physical activity**
**Local-IM**	1.00							
**Area-IM**	0.593 (0.001)	1.00						
**Pubertal Status**	0.125 (0.001)	0.128 (0.001)	1.00					
**BMI**	0.082 (0.003)	0.100 (0.001)	0.161 (0.001)	1.00				
**IMD**	0.022 (0.424)	0.111 (0.001)	0.112 (0.001)	0.126 (0.001)	1.00			
**Daylight**	0.143 (0.001)	0.115 (0.001)	0.059 (0.034)	0.006 (0.822)	0.100 (0.001)	1.00		
**Weekday physical activity**	0.180 (0.001)	0.188 (0.001)	-0.076 (0.008)	-0.089 (0.002)	0.110 (0.001)	0.243 (0.001)	1.00	
**Weekend physical activity**	0.112 (0.001)	0.092 (0.005)	-0.113 (0.001)	-0.061 (0.066)	0.065 (0.049)	0.108 (0.001)	0.430 (0.001)	1.00

BMI: body mass index

IMD:index of multiple deprivation

The results of the linear regression analysis are found in Table 4. In the unadjusted model, Local-IM was significantly (p < 0.01) associated with weekday and weekend physical activity in girls but only with weekday physical activity in boys. Area-IM was significantly related to weekday physical activity in both boys and girls. The positive association between Local-IM and Area-IM and weekday physical activity remained largely unchanged after adjustment for BMI, IMD, daylight and pubertal status. For weekend physical activity after adjustment, Local-IM remained significant in girls. The interaction of Local-IM by Area-IM was not significantly related to weekend or weekday physical activity for boys or girls.

**Table 4:** Unadjusted and adjusted cross-sectional associations between Local-IM, Area-IM and physical activity for boys and girls

	**Weekday physical activity (counts per minute)**				**Weekend physical activity (counts per minute)**			
**Boys**	**Beta** **(95% CI)**	**z**	***p***	**% Variance explained** **(within, between, overall)**	**Beta** **(95% CI)**	**z**	***p***	**% Variance explained** **(within, between, overall**)
Local-IM	33.55^a ^(19.23,47.87)	4.59	< 0.001	(0.032,0.144,0.041)	24.47^a^ (-2.319,51.25)	1.79	0.073	(0.007,0.009,0.010)
	35.92^b^ (21.59,50.24)	4.35	0.001	(0.029,0.071,0.038)	25.77^b^ (-1.63,53.17)	1.84	0.065	(0.030,0.216,0.045)
Area-IM	30.48^a^ (16.73,44.23)	4.35	0.001	(0.029,0.071,0.038)	21.92^a^ (-4.26,48.17)	1.64	0.101	(0.006,0.001,0.008)
	32.61^c^ (18.80,46.42)	4.63	< 0.001	(0.061,0.500,0.152)	25.42^c^ (-1.31,52.15)	1.86	0.062	(0.031,0.199,0.045)
Local-IM* Area-IM	6.51^d^ (-21.66,15.01)	0.60	0.547		8.40^d^ (-32.07,48.88)	0.41	0.684	

**Girls**	**Beta** **(95% CI)**	**Z**	***p***	% Variance explained (within, between, overall)	**Beta** **(95% CI)**	**z**	***p***	% Variance explained (within, between, overall)

Local-IM	17.89^a^ (6.20, 29.58)	3.00	0.003	(0.015,0.001,0.013)	24.13^a^ (4.49,43.78)	2.41	0.016	(0.005,0.226,0.011)
	16.88^b^ (4.98,28.79)	2.78	0.005	(0.021,0.239,0.053)	23.45^b^ (3.42,43.47)	2.29	0.022	(0.021,0.055,0.035)
Area-IM	21.03^a^ (8.43,33.64)	3.27	0.001	(0.016,0.095,0.024)	20.86^a^ (-0.589,42.31)	1.91	0.057	(0.002,0.179, 0.007)
	21.26^c^ (8.52,33.99)	3.27	0.001	(0.024,0.281,0.063)	18.93^c^ (-2.80,40.65)	1.71	0.088	(0.030,0.216,0.045)
Local-IM* Area-IM	3.31^d^ (-21.66,15.04)	-0.35	0.724		3.25^d^ (-28.87,35.37)	0.20	0.843	

^a ^Unadjusted model

^b ^Adjusted for BMI, IMD score, daylight, pubertal status

^c ^Adjusted for Local-IM, BMI, IMD score, daylight, pubertal status

^d ^Adjusted for Local-IM, Area-IM, BMI, IMD score, daylight, pubertal status

## Discussion

Two distinct subscales emerged to describe independent mobility in this population. They represent the frequency that children report being permitted to go to destinations unsupervised by an adult locally (Local-IM) and in the wider neighbourhood (Area-IM). As would be expected both boys and girls had higher scores for Local-IM than Area-IM indicating that parents were more willing to let them visit 'local' destinations i.e. friends house, park, local shops and school unsupervised compared to those which were assumed to be further away. Both Local-IM and Area-IM were higher in boys compared to girls. This is consistent with other studies [1,30] where parents appear more willing to let boys visit places outside the home unsupervised compared to girls.

Children who reported being allowed to visit destinations unsupervised locally (Local-IM) and in the wider (Area-IM) neighbourhood had higher levels of weekday physical activity compared to those who reported lower levels of Local-IM and Area-IM. This positive association with objectively measured physical activity for weekdays remained even after adjustment for a range of potential confounders (BMI, IMD, pubertal status and minutes of daylight from 3 pm until sunset). For weekend physical activity, only Local-IM in girls was significantly related to average weekend physical activity. The lack of significant association between Area-IM and weekend physical activity may indicate that at this age young people get the majority of their physical activity at weekends 'locally'. Logically however you would expect that young people have more time to visit destinations further afield at weekends. It may be that children at this age spend more time supervised by parents on weekend visits and as Area-IM scores are hypothesised to only reflect 'unsupervised' visits, Area-IM is not associated with weekend physical activity. It is possible that Area-IM may relate to physical activity at weekends later in adolescence as distance travelled and range of destinations visited unsupervised increases [1]. Variability in Area-IM scores is also less than that for Local-IM so could have affected power to detect significant relationships in analyses.

The assumption that Local-IM reflects destinations accessible to young people unsupervised and that Area-IM reflects destinations further away from where children live needs to be confirmed. Objective measures such as Geographical Information Systems could be used alongside self-reported independent mobility to ascertain the location of destinations visited unsupervised.

Physical activity and factors such as independent mobility are likely to be influenced by the type of neighbourhood (housing density, land use mix, available green space) as well as perceptions of that neighbourhood. For example, a parent may be much more likely to allow independent mobility if they perceive their environment to be safe and traffic density to be low and vice versa. Although the current study includes a wide range of participants from a single city, the variability in types of neighbourhood within a city could be smaller than that between different cities. These findings therefore should be confirmed in other geographical locations. The inclusion of minutes of daylight after school (3 pm) is relatively unusual in the literature, but these data confirm that minutes of daylight are positively related to both independent mobility and physical activity so should be considered as a potential confounder in future work. Available daylight is particularly relevant when investigating independent mobility as darkness has been reported as a barrier to parents allowing their children to play outside unsupervised [1]. Studies where the measurement period is restricted to only winter or summer may not therefore be generalisable to other seasons.

Our limited knowledge with respect to independent mobility and physical activity may be a result of measurement challenges and lack of a coherent theoretical framework. Many studies including this one have relied on self-reported perceptions of independent mobility by either the child or the parent. Where direct measures such as detailed observation have been used, samples are usually relatively small and often in restricted situations (e.g. close to home or in the school playground). New equipment taking advantage of developments in satellite technology (e.g. portable Global Positioning Satellite (GPS) receivers) offer the potential for novel objective measures of children's movement within their neighbourhood. If combined with accelerometry data and information on level of supervision they offer a potentially more robust approach to measuring independent mobility in relation to physical activity. Examples of this integration of measures are slowly emerging in the literature [32]. Data presented are to date based on small, homogeneous samples from a narrow range of neighbourhoods and reliability and validity of output generated by GPS and accelerometry has rarely been fully described [33]. Larger more diverse studies are required that can document movement within the environment for a broad range of children and neighbourhoods.

We know very little about the factors which determine level of independent mobility in young people. Johansson [34] compared the characteristics of parents whose attitudes favour car use compared to those who favour independent travel. They found that parents with a favourable attitude towards independent travel were more likely to express a strong trust in the environment and road users and felt less need to protect their children. We also know that older children and males experience more independent mobility than females and younger children and that the reasons for decisions related to independent mobility in young people are a complex interaction between the child, the family and the environment [13].

This complexity can only be addressed by a coherent theoretical framework where independent mobility is combined with other more established physical, social-environmental and personal determinants of physical activity [35]. A limitation of this paper is its lack of theoretical direction. Although a rationale for investigating independent mobility in relation to physical activity has been presented the independent mobility constructs developed have not been systematically linked to other determinants of physical activity.

Other constructs that relate to independent mobility can be found within existing theoretical frameworks. Examples of these constructs include autonomous motivation embedded in the Self Determination Theory [36,37] and habits and norm directed behaviour in relation to humans and the environment [38-40]. However there has been limited theoretical work focussed specifically on the concept of independent mobility and physical activity. A greater emphasis on IM is consistent with the shift in recent years to focus on changing the environment as well as the individual as part of the public health agenda [41]. However the cross-sectional nature of this study means we cannot determine whether higher levels of independent mobility are causally linked to higher levels of physical activity or vice versa. Longitudinal data are required to establish whether change in independent mobility is associated with a concomitant change in physical activity.

The main limitations of this study are the reliance on self-reported independent mobility and the cross-sectional design. The strengths of this study include the development of a specific measure of independent mobility, the use of an objective measure of physical activity in a relatively large sample and the measurement of and adjustment for a range of confounders in the analyses.

## Conclusion

The decline in children's independent mobility and the potential negative consequences this may have not only for physical activity levels but also broader physical and social well being means this concept merits more systematic inclusion in the determinants literature. In this study we have found that greater levels of Local-IM and Area-IM were associated with greater volume of physical activity on weekdays. Understanding the factors that influence independent mobility is necessary to determine the optimum social and physical environment that encourages parents and adult carers to allow their children to be physically active outside unsupervised. This should be in addition to encouraging children (and parents) to be more physically active outside together. Both of these approaches may be important mechanisms to promote increased physical activity in young people.

## Competing interests

The authors declare that they have no competing interests.

## Authors' contributions

AP drafted the initial manuscripts and conducted the analyses. All other authors contributed to the design of the project and the writing of the manuscript. All authors read and approved the final manuscript.
